# Metformin Alleviates Stress-Induced Premature Senescence of Vascular Endothelial Cells by Regulating Mitocytosis

**DOI:** 10.3390/ijms27114724

**Published:** 2026-05-24

**Authors:** Hui Lu, Qing Mu, Boqun Wang, Yan Chen, Binghui Zeng, Lisha Gu, Wei Zhao

**Affiliations:** Guanghua School of Stomatology, Hospital of Stomatology, Guangdong Provincial Key Laboratory of Stomatology, Sun Yat-sen University, Guangzhou 510055, China; luhui25@mail.sysu.edu.cn (H.L.); muqing3@mail2.sysu.edu.cn (Q.M.);

**Keywords:** metformin, stress-induced premature senescence, mitocytosis, vascular endothelial cells

## Abstract

Stress-induced premature senescence (SIPS) of endothelial cells can cause endothelial dysfunction. As a first-line antidiabetic agent, the specific role of metformin in SIPS has not yet been clarified. In this study, an in vitro SIPS model was induced by exposing human umbilical vein endothelial cells (HUVECs) to hydrogen peroxide (H_2_O_2_), and the effects of metformin on cell senescence, proliferation, migration, tube formation, and mitochondrial function were evaluated. Gene expressions altered by metformin were profiled via transcriptome sequencing. Specifically, the potential involvement of migrasome-mediated mitocytosis in metformin-driven effects was examined using confocal microscopy and siRNA-mediated silencing. The results showed that metformin significantly reduced SA-β-gal activity and restored the migration and tube-forming capacities of H_2_O_2_-induced senescent HUVECs. Moreover, metformin regulated mitochondrial dynamics, restored mitochondrial membrane potential, and attenuated intracellular oxidative stress. Notably, transcriptomic and functional analyses suggested that metformin enhanced migrasome formation and migrasome-mediated mitocytosis. Inhibition of migrasome formation by siTSPAN4 abolished the protective effect of metformin against SIPS. Collectively, these findings demonstrate that metformin alleviates early SIPS-associated changes in HUVECs and suggest that migrasome-mediated mitocytosis contributes to this protection by ameliorating mitochondrial dysfunction. This provides novel mechanistic insight into the vascular protective effects of metformin.

## 1. Introduction

The proper function of vascular endothelial cells is indispensable for maintaining vascular homeostasis in diabetes and cardiovascular disease. These cells regulate vascular tension, promote angiogenesis, and participate in inflammatory responses. However, exposure to oxidative stress, inflammation, or high glucose can induce endothelial dysfunction. Among the underlying pathological processes, stress-induced premature senescence (SIPS) has emerged as a key contributor [1,2]. As an early feature of cardiovascular disease, SIPS is marked by reduced proliferative capacity, elevated senescence markers, increased oxidative stress, and impaired vascular diastolic function [3,4]. It compromises endothelial barrier integrity, increases vascular permeability, and disrupts vascular homeostasis, making strategies that inhibit SIPS and restore endothelial function therapeutically promising.

Metformin is a first-line drug for type 2 diabetes which is well known for its glucose-lowering effects. Beyond glycemic control, it exerts multiple biological effects including metabolic regulation, antioxidant activity, immunomodulation, and osteogenic potential, primarily acting through the activation of AMP-activated protein kinase (AMPK) [5,6,7,8]. Metformin also reduces mitochondrial oxidative stress and improves mitochondrial function, which are crucial for cellular homeostasis [9,10]. It has been shown that metformin attenuates endothelial impairment and suppresses senescence, thereby preserving endothelial function and slowing cardiovascular disease progression [9,11,12]. It can also promote tube formation under hyperglycemic/hypoxic conditions [13]. However, the specific mechanism by which metformin alleviates vascular endothelial SIPS—particularly its potential role in mitochondrial homeostasis—has not been fully clarified.

Mitochondrial dysfunction is a key driver of SIPS, compromising vascular stability and promoting endothelial dysfunction [14]. Mitochondrial quality control sustains mitochondrial homeostasis by governing mitochondrial dynamics (fission and fusion), intracellular mitochondrial movement, selective removal of damaged mitochondria through mitophagy, and mitochondrial biogenesis [15,16]. Notably, mitocytosis, first identified in 2021, represents a novel migrasome-mediated mitochondrial quality control mechanism, wherein cells expel damaged mitochondria or mitochondrial fragments via membrane fusion and extracellular release [17]. Unlike canonical mitophagy, which degrades mitochondria within lysosomes, mitocytosis physically ejects dysfunctional mitochondria, thereby preserving mitochondrial network integrity. Endothelial cells possess strong migratory capacity and form retraction fibers and migrasomes, which are critical for mitocytosis. Senescent cells show impaired migratory capacity and reduced migrasome formation [18], resulting in defective mitocytosis [19]. Considering that metformin has been shown to regulate endothelial cell migration [20], we sought to determine whether the beneficial effects of metformin in vascular endothelial cells are linked to migrasome-mediated mitocytosis.

This study explores the therapeutic potential of metformin in a H_2_O_2_-induced premature senescence model of human umbilical vein endothelial cells (HUVECs) in vitro, with a focus on the regulatory role of mitocytosis. Our results suggest that metformin alleviates oxidative stress-induced injury and enhances angiogenesis, at least in part, by restoring mitochondrial homeostasis through migrasome-mediated mitocytosis. These findings support the therapeutic potential of metformin in addressing stress-induced vascular pathologies and suggest migrasome-mediated mitocytosis as a potential mechanism for maintaining vascular homeostasis, indicating a promising therapeutic target for endothelial dysfunction linked to SIPS.

## 2. Results

### 2.1. Metformin Alleviates H_2_O_2_-Induced Stress-Induced Premature Senescence in HUVECs

**Figure 1:**
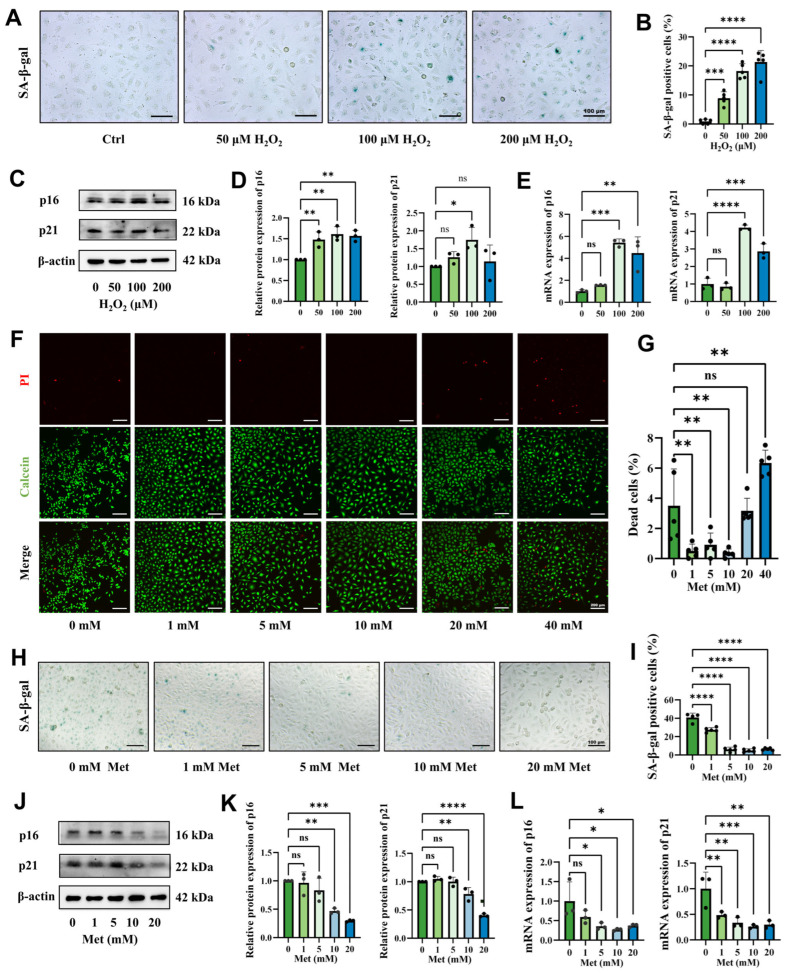
Metformin eliminated stress-induced premature senescence of HUVECs induced by 100 μM H_2_O_2_. (**A**,**B**) Representative images and quantification of senescence-associated β-galactosidase (SA-β-gal)-positive HUVECs under induction with different concentrations of H_2_O_2_ (scale bar: 100 μm). (**C**,**D**) Western blot analysis for the senescence markers p16 and p21 under induction with different concentrations of H_2_O_2_. (**E**) Relative mRNA expression levels of senescence markers p16 and p21 under induction with different concentrations of H_2_O_2_. (**F**,**G**) Live/Dead staining and quantification of HUVECs treated with different concentrations of metformin. Live cells and dead cells were labeled with Calcein (green) and PI (red), respectively (scale bar: 200 μm). (**H**,**I**) Representative images and quantification of SA-β-gal-positive HUVECs treated with different concentrations of metformin (scale bar: 100 μm). (**J**,**K**) Western blot analysis for the senescence markers p16 and p21 treated with different concentrations of metformin. (**L**) Relative mRNA expression levels of senescence markers p16 and p21 treated with different concentrations of metformin. Data are presented as the mean ± standard deviation. * *p* < 0.05, ** *p* < 0.01, *** *p* < 0.001, and **** *p* < 0.0001. ns, no significance.

As oxidative damage is a primary driver of SIPS in HUVECs, we established an H_2_O_2_-based model to investigate metformin’s protective effects. Cellular senescence was validated through senescence-associated β-galactosidase (SA-β-gal) staining, qRT-PCR, and Western blot analyses. SA-β-gal staining revealed a concentration-dependent increase in positive cells with rising H_2_O_2_ levels (Figure 1A,B). Notably, treatment with 100 μM H_2_O_2_ for 2 h induced the most pronounced upregulation of senescence-related markers (p16 and p21), as confirmed via Western blot and qRT-PCR (Figure 1C,D). A similar trend was observed at the mRNA level (Figure 1E). Accordingly, 100 μM H_2_O_2_ was selected to induce SIPS in HUVECs for subsequent experiments.

To assess metformin’s effect on the viability of HUVECs under oxidative stress, a Live/Dead assay was performed. Quantitative analysis revealed that 1–10 mM metformin significantly decreased the proportion of dead cells (stained with red) compared to the H_2_O_2_-treated control (*p* < 0.01), with cell death remaining minimal (<2%). At 20 mM, metformin exerted no significant effect on viability. However, 40 mM metformin increased the percentage of dead cells (*p* < 0.01), suggesting dose-dependent cytotoxicity at elevated concentrations (Figure 1F,G). Consequently, metformin concentrations of 0–20 mM were employed in subsequent senescence assays to avoid cytotoxic influences.

Subsequently, SA-β-gal staining was performed to evaluate the effect of metformin on H_2_O_2_-induced premature senescence. The results showed that metformin attenuated H_2_O_2_-induced senescence in a dose-dependent manner (Figure 1H,I). Compared to the H_2_O_2_-treated control, metformin decreased the proportion of SA-β-gal-positive cells with increasing dose (*p* < 0.001). Consistent with these findings, Western blot analysis revealed that metformin dose-dependently downregulated p16 and p21 expression, which was raised by H_2_O_2_ induction (Figure 1J,K). Significant reductions were observed at 10 mM (*p* < 0.01) and 20 mM (*p* < 0.001). At the mRNA level, 10 mM metformin significantly inhibited the expression of these senescence markers, corroborating the protein results (Figure 1L). Collectively, these results demonstrate that metformin ameliorates H_2_O_2_-induced SIPS in HUVECs with optimal efficacy and safety observed at 10 mM, a concentration that does not adversely impact cell viability.

### 2.2. Metformin Promotes Migration and Angiogenesis of HUVECs with SIPS

**Figure 2:**
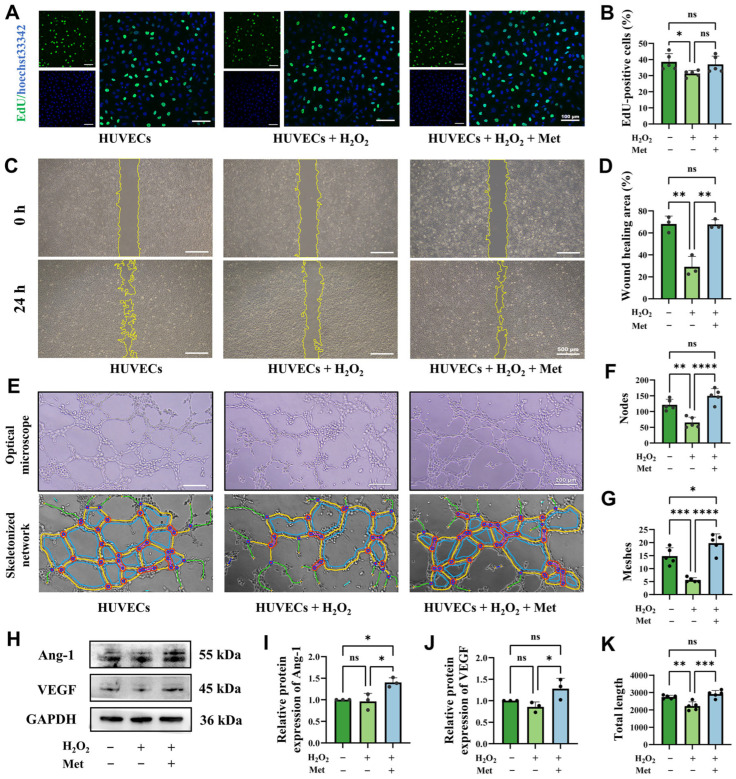
Effects of metformin on cell proliferation, migration, and angiogenic potential of HUVECs. (**A**,**B**) Representative EdU images and quantification analysis showing proliferation capacity of HUVECs treated with metformin (scale bar: 100 μm). (**C**) Representative images of scratch wound healing monitored for 24 h (scale bar: 500 µm). (**D**) Quantification of wound healing areas. (**E**–**G**,**K**) Tube formation analysis of the angiogenesis of HUVECs treated with metformin (scale bar: 200 μm). (**H**–**J**) Western blot analysis of VEGF and Ang-1 in HUVECs treated with metformin. * *p* < 0.05, ** *p* < 0.01, *** *p* < 0.001, and **** *p* < 0.0001. ns, no significance.

Cell proliferation was assessed via an EdU assay (Figure 2A). H_2_O_2_ exposure significantly reduced the proportion of EdU-positive cells from 38.53% in the control group to 31.25% (*p* < 0.05). Metformin treatment increased this proportion to 37%, although no significant difference was revealed (*p* > 0.05, Figure 2B). Regarding cell migration, scratch wound healing assays revealed that metformin enhanced HUVEC migration compared to the H_2_O_2_-treated group. H_2_O_2_-induced SIPS severely delayed wound healing, with only 29% of the wound area closed at 24 h, while near-complete wound closure was observed in both the healthy control and metformin-treated groups at the same time point (Figure 2C,D).

Tube formation assay further validated metformin’s pro-angiogenic effects. Upon stimulation with H_2_O_2_, tube formation was compromised, manifesting as less well-organized vascular-like structures and reduced node numbers. Metformin effectively restored the angiogenic capacity of HUVECs that were compromised by H_2_O_2_ exposure (Figure 2E). Compared to the H_2_O_2_-treated group, metformin elicited an elevation in tube nodes (*p* < 0.0001, Figure 2F), meshes (*p* < 0.0001, Figure 2G), and total tube length (*p* < 0.001, Figure 2K). Consistent with these results, Western blot analysis confirmed upregulated expression of angiogenesis-related proteins. The levels of Ang-1 and VEGF were significantly elevated in metformin-treated cells relative to the H_2_O_2_-treated group (*p* < 0.05, Figure 2H–J). Together, these findings establish that metformin attenuated ROS damage while promoting migration and angiogenesis in HUVECs under SIPS.

### 2.3. Metformin Mitigates Mitochondrial Dysfunction in HUVECs

**Figure 3:**
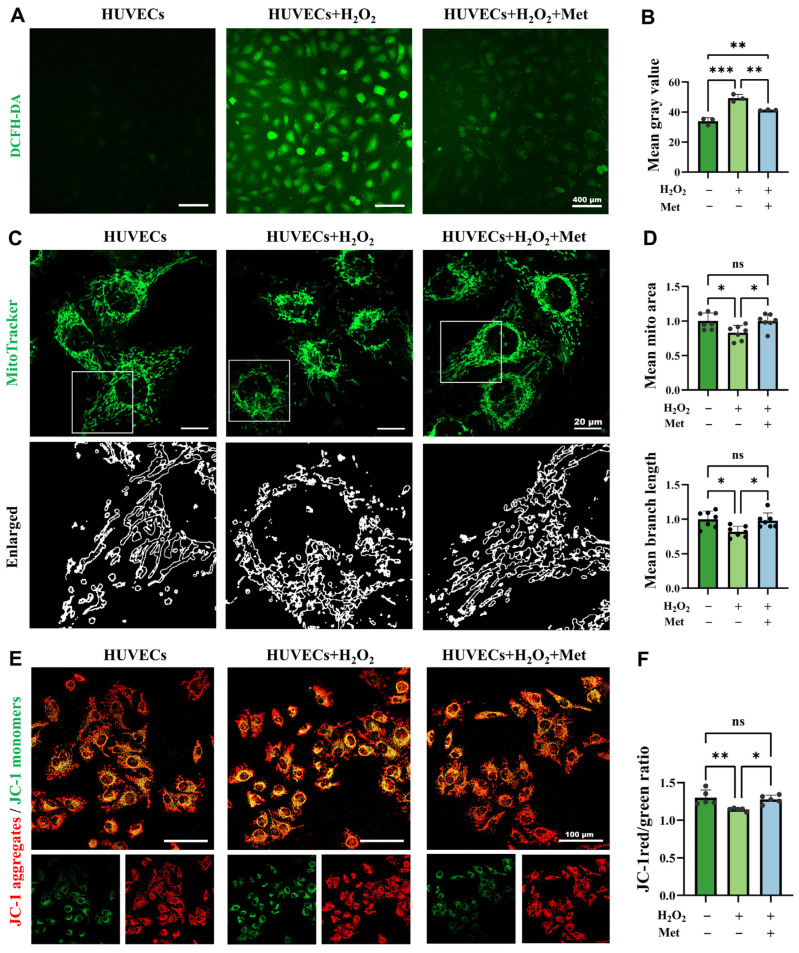
Metformin preserved mitochondrial homeostasis in HUVECs. (**A**,**B**) Representative immunofluorescence images and quantification showing intracellular ROS levels in HUVECs (scale bar: 400 μm). (**C**) Mitochondrial morphology was visualized using MitoTracker staining (scale bar: 20 μm). (**D**) Quantitative analysis of mitochondrial area and length based on MitoTracker staining. (**E**) Representative images showing mitochondrial membrane potential (MMP) assessed using JC-1 fluorescent probes. JC-1 aggregates were shown in red, and JC-1 monomers were shown in green (scale bar: 100 μm). (**F**) Analysis of the JC-1 red/green fluorescence ratio. Data are presented as the mean ± standard deviation. * *p* < 0.05, ** *p* < 0.01, and *** *p* < 0.001. ns, no significance.

To assess metformin’s effects on oxidative stress, intracellular reactive oxygen species (ROS) levels were quantified using DCFH-DA. The results revealed a significant elevation in ROS levels following H_2_O_2_ treatment (*p* < 0.001), whereas metformin attenuated this increase (*p* < 0.01), suggesting that metformin effectively inhibits H_2_O_2_-induced ROS overproduction and alleviates oxidative stress (Figure 3A,B). Given the close association between ROS-induced damage and mitochondrial dysfunction, the influence of metformin on mitochondrial homeostasis was subsequently assessed.

Mitochondrial morphology and distribution were examined through MitoTracker (Figure 3C,D). In healthy HUVECs, mitochondria exhibited a uniform, filamentous structure with regular intracellular distribution. In contrast, exposure to H_2_O_2_ induced mitochondrial fragmentation, irregular perinuclear aggregation, and disrupted network integrity, which are typical features of mitochondrial dysfunction. Metformin treatment preserved mitochondrial morphology, as manifested by increased mitochondrial areas and enlarged branch length (*p* < 0.05). Mitochondrial membrane potential (MMP), a key indicator of functional integrity, was assessed using JC-1 fluorescent probes. H_2_O_2_ exposure significantly reduced the JC-1 red/green fluorescence ratio, indicating MMP depolarization and compromised mitochondrial function. Conversely, metformin intervention notably increased this ratio in H_2_O_2_-treated cells (Figure 3E,F). These findings indicate that metformin alleviates H_2_O_2_-induced mitochondrial dysfunction in HUVECs by inhibiting excessive ROS generation and restoring MMP.

### 2.4. Transcriptomic Characteristics of Metformin-Treated HUVECs Under SIPS

**Figure 4:**
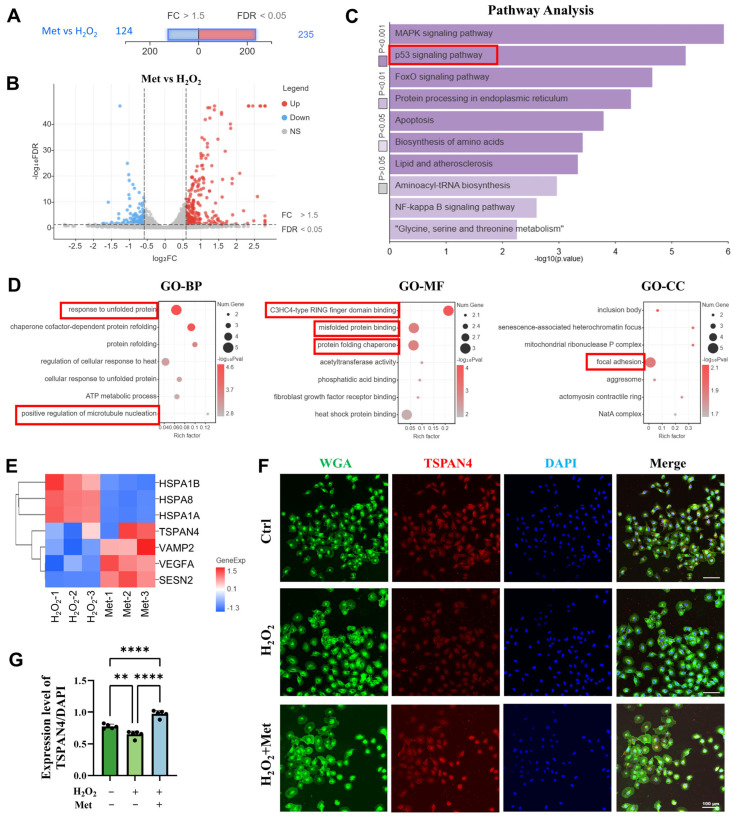
Transcriptomic sequencing analysis reveals the role of migrasome-mediated mitocytosis mediated by metformin. (**A**,**B**) DEGs of the metformin group compared to the H_2_O_2_ group were displayed in a histogram and a volcano plot (FDR < 0.05, |FC| > 1.5). Note: Met, HUVECs treated with H_2_O_2_ and metformin; H_2_O_2_, HUVECs treated with H_2_O_2_ only. (**C**) KEGG analysis of DEGs. (**D**) GO analysis of upregulated genes in the metformin group compared to the H_2_O_2_ group on the basis of biological processes (BP), molecular function (MF), and cellular component (CC). (**E**) Representative differential genes between the H_2_O_2_ and Met group. (**F**,**G**) Representative immunofluorescent images of TSPAN4 in HUVECs post H_2_O_2_-treatment with and without metformin (scale bar: 100 μm). Data are presented as the mean ± standard deviation. ** *p* < 0.01, and **** *p* < 0.0001.

To profile transcriptional changes associated with metformin-induced restoration in H_2_O_2_-treated HUVECs, RNA sequencing was performed to compare the H_2_O_2_ group (H_2_O_2_-treated) and the Met group (H_2_O_2_-treated + metformin). A total of 235 upregulated and 124 downregulated differentially expressed genes (DEGs) were identified in the metformin-treated group, with the criteria of false discovery rate (FDR) < 0.05 and |fold change (FC)| > 1.5 (Figure 4A,B). KEGG pathway enrichment analysis revealed that the DEGs were significantly enriched in signaling pathways related to cellular senescence, stress response, and vascular homeostasis, including the MAPK, p53, and FoxO signaling pathways (Figure 4C). Gene Ontology (GO) analysis annotated the DEGs from the perspectives of biological process (BP), molecular function (MF), and cellular component (CC). The results showed that the DEGs were enriched in functional terms linked to protein homeostasis and stress response, including misfolded protein binding, protein folding chaperone complex, and heat shock protein binding, as well as terms related to cell adhesion and cytoskeleton regulation, such as focal adhesion and positive regulation of microtubule nucleation (Figure 4D). Expression heatmaps of genes implicated in protein homeostasis and stress response are presented in Figure 4E. Metformin significantly downregulated the expression of heat shock protein family genes (HSPA1B, HSPA8, HSPA1A) associated with cellular stress and protein homeostasis, while enhancing the expression of SESN2, a key gene involved in oxidative stress response and mitochondrial homeostasis [21]. Concurrently, upregulated expression of VEGFA was also observed. Most importantly, metformin upregulated the transcription levels of vesicle-associated membrane protein 2 (VAMP2) and tetraspanin 4 (TSPAN4), both of which are closely associated with migrasome formation and mitocytosis. To corroborate the expression of TSPAN4, a pivotal protein for migrasome biogenesis, immunofluorescence staining was performed. The results confirmed that TSPAN4 was upregulated in the metformin-treated group, with an increase of 1.43-fold (Figure 4F,G), consistent with the transcriptomic results.

### 2.5. Metformin Promotes Migrasome Formation and Migrasome-Mediated Mitocytosis

**Figure 5:**
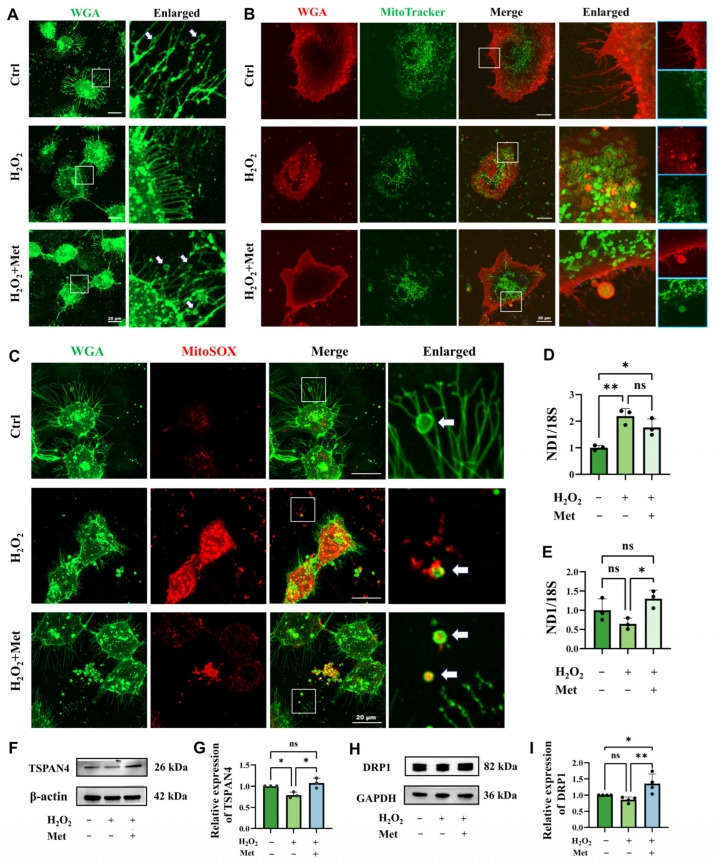
Metformin facilitated migrasome formation and dysfunctional mitochondrial expulsion in a migrasome-regulated manner. (**A**) Representative images showing the formation of migrasomes. (scale bar: 20 μm). White arrows indicate the migrasome. (**B**) Representative confocal images of HUVECs stained with WGA (red) and MitoTracker (green) (scale bar: 20 μm). (**C**) Confocal images of HUVECs stained with WGA (green) and MitoSOX (red) (scale bar: 20 μm). White arrows indicate the migrasome. (**D**) Quantification of mtDNA in the culture medium. (**E**) Quantification of mtDNA in the isolated migrasomes. (**F**–**I**) Western blot analysis for proteins associated with migrasome formation. Data are presented as the mean ± standard deviation. * *p* < 0.05, and ** *p* < 0.01. ns, no significance.

To directly verify the functional role of migrasomes and migrasome-mediated mitocytosis, cells were stained with wheat germ agglutinin (WGA), a specific probe for migrasome detection. Compared to the control group, H_2_O_2_ exposure caused retraction fiber shortening and reduced migrasome formation. In contrast, metformin treatment markedly reversed the inhibitory effect of H_2_O_2_ on migrasome biogenesis (Figure 5A). To further confirm whether metformin-promoted migrasomes are involved in mitocytosis, we co-stained HUVECs with WGA and MitoTracker. The results showed that fragmented mitochondria accumulated around the cell membrane in the H_2_O_2_-treated group, while metformin enhanced the colocalization of mitochondria and migrasome signals, with mitochondria gathering inside the migrasome structures (Figure 5B), indicating that metformin promotes the recruitment of mitochondria to migrasomes. Moreover, mitochondrial status within migrasomes was detected using MitoSOX (Figure 5C). Compared to the control group, H_2_O_2_ treatment obviously caused the accumulation of intracellular MitoSOX signal, accompanied by numerous damaged mitochondria scattered outside cells. However, metformin decreased the intracellular MitoSOX signal, and a significantly elevated MitoSOX signal was observed in migrasomes. Furthermore, mtDNA was detected via qRT-PCR to assess the amounts of extracellular mitochondria. The results showed that the relative mtDNA levels in culture medium were obviously elevated in the H_2_O_2_ group and H_2_O_2_ + Met group compared to the control group (*p* < 0.01 and *p* < 0.05, respectively), whereas no significant difference was found between the H_2_O_2_ group and H_2_O_2_ + Met group (Figure 5D). Quantification of mtDNA in isolated migrasomes revealed a modest but significant increase in ND1/18S in the H_2_O_2_ + Met group compared to the H_2_O_2_ group (*p* < 0.05, Figure 5E), suggesting that metformin promoted the packaging of damaged mitochondria into migrasomes.

Additionally, Western blot analysis was performed to detect the expression of key proteins related to migrasome formation and mitochondrial dynamics (Figure 5F–I). Metformin treatment resulted in a significant upregulation of TSPAN4 (*p* < 0.05), a core structural protein essential for migrasome biogenesis. Meanwhile, metformin also increased the expression of dynamin-related protein 1 (DRP1) (*p* < 0.01), which is critical for mitochondrial intracellular transport. These findings indicate that metformin not only promotes migrasome formation but also modulates mitochondrial dynamics, thereby coordinately facilitating mitocytosis.

### 2.6. Metformin-Enhanced Mitocytosis Is Largely Independent of AMPK and Mitophagy

**Figure 6:**
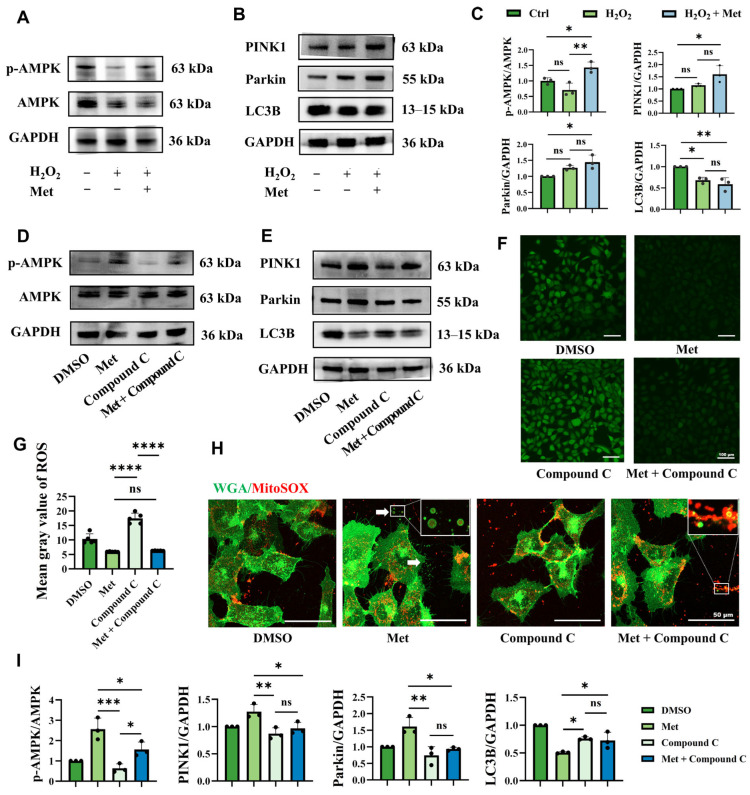
Metformin enhances mitocytosis in a manner largely independent of AMPK and mitophagy. (**A**–**C**) Western blot and relative quantitative analysis of AMPK signaling markers and mitophagy markers. (**D**,**E**,**I**) Western blot analysis for proteins associated with AMPK signaling and mitophagy in HUVECs treated with or without Compound C. (**F**,**G**) Representative immunofluorescence images and quantification showing intracellular ROS levels in HUVECs treated with or without Compound C (scale bar: 100 μm). (**H**) Confocal images of HUVECs stained with WGA (green) and MitoSOX (red) (scale bar: 50 μm). White arrows indicate the migrasome. Data are presented as the mean ± standard deviation. * *p* < 0.05, ** *p* < 0.01, *** *p* < 0.001, and **** *p* < 0.0001. ns, no significance.

To determine whether the observed effects of metformin are mediated by canonical AMPK signaling and mitophagy, the protein levels associated with AMPK activation and mitophagy were first examined via Western blot. As shown in Figure 6A–C, metformin treatment increased the levels of phosphorylated AMPK (p-AMPK) compared to the control, indicating AMPK activation under our experimental conditions. Concurrently, increased expression of PINK1 and Parkin, and decreased expression of LC3B were observed in the metformin-treated group. Pretreatment with the AMPK inhibitor Compound C effectively blocked metformin-induced p-AMPK elevation and partially reversed the metformin-induced changes in PINK1, Parkin, and LC3B expression (Figure 6D,E,I). However, no significant difference in ROS reduction was observed between the Met group and the Met + Compound C group (Figure 6F,G). In terms of MitoSOX and migrasome staining, Compound C treatment led to an increase in extracellular MitoSOX-positive mitochondria, whereas migrasome formation remained detectable (Figure 6H). Collectively, these data suggest that while metformin activates AMPK signaling and modulates mitophagy-related proteins, the migrasome-mediated mitocytosis phenotype appears to be largely independent of AMPK activity.

### 2.7. Inhibition of Migrasome Formation by siTSPAN4 Impacts Metformin Effects

**Figure 7:**
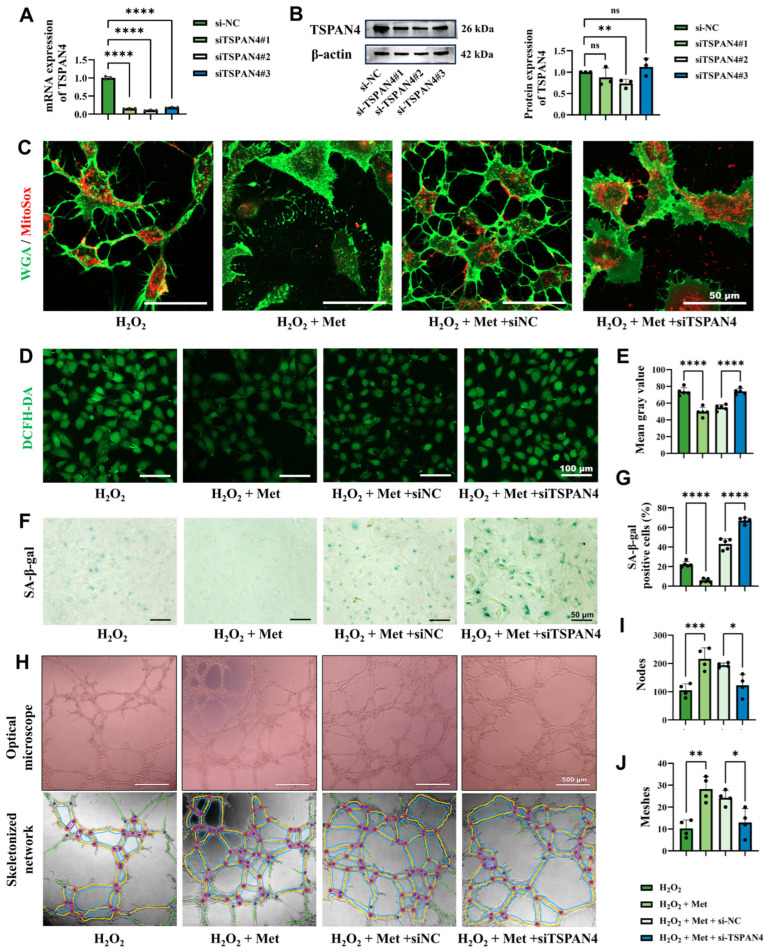
Inhibition of migrasome formation by siTSPAN4 impacts metformin effects. (**A**,**B**) qRT-PCR and Western blot indicated that siTSPAN4#2 exhibits the highest knockdown efficiency compared to siNC. (**C**) Representative confocal images of HUVECs stained with WGA and MitoSOX (scale bar: 50 μm). (**D**,**E**) Representative immunofluorescence images and quantification showing intracellular ROS levels in HUVECs (scale bar: 100 μm). (**F**,**G**) Representative images and quantification of SA-β-gal-positive HUVECs treated with different concentrations of metformin (scale bar: 50 μm). (**H**–**J**) Tube formation analysis of the angiogenesis of HUVECs (scale bar: 500 μm). Data are presented as the mean ± standard deviation. * *p* < 0.05, ** *p* < 0.01, *** *p* < 0.001 and **** *p* < 0.0001. ns, no significance.

To validate the pivotal role of migrasome-mediated mitocytosis in the mechanism underlying metformin’s efficacy, HUVECs were transfected with siTSPAN4 to suppress migrasome formation. Three specific siRNA sequences targeting TSPAN4 (si-TSPAN4#1, si-TSPAN4#2, si-TSPAN4#3) and a negative control siRNA (si-NC) were employed, and the knockdown efficiency was confirmed through qRT-PCR and Western blot analyses (Figure 7A,B). Based on the validation results, si-TSPAN4#2 was selected for subsequent experiments.

After cell transfection and H_2_O_2_ stimulation, migrasome formation in HUVECs was examined via confocal laser scanning microscope (CLSM) using WGA (green) and MitoSOX (red). The results revealed that TSPAN4 knockdown (Met + siTSPAN4 group) significantly inhibited retraction fiber formation and migrasome biogenesis, accompanied by a marked increase in MitoSOX levels (Figure 7C). Changes in intracellular ROS levels were detected by DCFH-DA. As shown in Figure 7D,E, metformin significantly reduced intracellular ROS accumulation compared to the H_2_O_2_-exposed control, while TSPAN4 knockdown in metformin-treated HUVECs led to a significant rebound of intracellular ROS levels (*p* < 0.0001), suggesting that intracellular ROS accumulation resulted from the suppression of migrasome-mediated mitocytosis.

SA-β-gal staining was subsequently performed to evaluate alterations in cellular senescence (Figure 7F,G). Compared to the si-NC group, the number of SA-β-gal-positive cells in the Met + siTSPAN4 group was significantly increased (*p* < 0.0001), suggesting that TSPAN4 knockdown reversed the anti-senescence effect of metformin. Moreover, tube formation assays were performed to assess angiogenic capacity, with node and mesh numbers quantified (Figure 7H–J). TSPAN4 knockdown abrogated metformin’s pro-angiogenic effect, leading to decreases in node and mesh numbers (*p* < 0.05). Taken together, these results indicate that inhibition of migrasome formation by siTSPAN4 abolishes the protective effects of metformin against SIPS in HUVECs, encompassing the reduction in oxidative stress, alleviation of cellular senescence, and enhancement of tube formation capacity.

## 3. Discussion

SIPS of vascular endothelial cells is a pivotal initiating event in the pathogenesis of cardiovascular diseases. As a classic antidiabetic agent, metformin has garnered extensive attention for its pleiotropic effects beyond glucose control, including anti-aging, antioxidative, and vascular protective properties [22,23,24]. However, the effects and mechanism by which metformin attenuates endothelial SIPS remain insufficiently defined. In the present study, we demonstrated that metformin effectively alleviates early SIPS-associated changes and restores angiogenic capacity in HUVECs, at least in part, by maintaining mitochondrial homeostasis via migrasome-mediated mitocytosis. These findings identify mitocytosis as a potential mechanism underlying metformin-driven vascular protection under oxidative stress and provide novel insights into the functional role of mitocytosis in endothelial SIPS.

H_2_O_2_ stimulation is widely used to induce oxidative stress and model SIPS [25,26]. In this study, HUVECs were treated with 100 μM H_2_O_2_ for 2 h to induce SIPS. After 24 h, EdU staining, SA-β-gal staining, and the expression levels of p21 and p16 were detected to evaluate oxidative stress-induced alterations in HUVECs [27,28]. The results showed that the EdU-positive rate in H_2_O_2_-treated HUVECs decreased from 38.5% to 31.3%, and SA-β-gal positivity increased to about 20%. Typically, the full SIPS phenotype requires 3–5 days to develop stable cell cycle arrest, sustained SA-β-gal positivity, p16/p21 upregulation, and cell shape change. Our data indicated that the cellular state at 24 h represented a mixture of acute oxidative injury, early senescence initiation, and partial residual proliferative activity, rather than a pure, established SIPS phenotype. Accordingly, this study focuses primarily on early SIPS-associated changes caused by oxidative stress.

Oxidative stress is the key factor triggering SIPS and causing endothelial dysfunction [2]. Accumulating evidence has confirmed that metformin can alleviate ROS production, enhance antioxidant defenses, and improve mitochondrial function, thereby enhancing cellular adaptability to oxidative stress [29]. In a H_2_O_2_-induced injury model of human periodontal ligament cells, metformin preconditioning reduced the expression of senescence-related genes and restored stemness [30], supporting the therapeutic potential of metformin in SIPS. In the present study, given the potential dose-dependent effects of metformin [31,32], we initially assessed the cytotoxic profile of metformin in HUVECs. Our results demonstrated that 10 mM metformin exerted no significant adverse effects on cell viability, which is consistent with previous studies [20,33]. Subsequent phenotypic assays showed that metformin not only diminished SA-β-gal activity and downregulated the expression of senescence-related genes, but also restored the migratory and tube-forming abilities of HUVECs with SIPS, thereby reversing endothelial dysfunction. Concurrently, metformin stabilized the mitochondrial membrane potential and regulated mitochondrial morphological dynamics, implying that the restoration of mitochondrial homeostasis is critically involved in metformin-mediated endothelial protection.

Mitochondria are highly dynamic and multifunctional organelles, and play an indispensable role in bioenergetics and cellular homeostasis. The preservation of mitochondrial homeostasis hinges on rigorous mitochondrial quality control, a process meticulously orchestrated through the regulation of mitochondrial dynamics, trafficking, and the selective clearance of damaged mitochondria [34,35]. It is well known that metformin targets mitochondria primarily by inhibiting mitochondrial complex I in the mitochondrial respiratory chain and suppressing glycerophosphate dehydrogenase [36,37,38]. In addition, mitochondrial dynamics, regulated by key proteins including DRP1, MFN1/2, and OPA1, actively participate in mediating the regulatory effects of metformin [39,40]. Substantial evidence places mitochondria at the center of metformin’s biological effects [41]. In the present study, we demonstrated that metformin not only reversed H_2_O_2_-induced mitochondrial fragmentation and the dissipation of mitochondrial membrane potential, but also concomitantly reduced intracellular ROS and mtROS levels. Meanwhile, we noted that fragmented mitochondria accumulated extensively in the perinuclear region under oxidative stress, while metformin treatment effectively reversed this abnormal distribution. These findings suggest that, beyond mitochondrial bioenergetics, mitochondrial trafficking may also contribute to the restoration of mitochondrial homeostasis under SIPS.

Migrasomes are newly identified organelles formed during cell migration, with diameters ranging from 0.5 to 3 μm. Their biogenesis is regulated by tetraspanins (TSPANs), cholesterol, and integrins [42]. They carry diverse cellular contents which have been implicated in multiple physiological and pathological processes [43]. As an important cargo of migrasomes, damaged mitochondria can be transported into migrasomes and subsequently discarded from migrating cells under mild mitochondrial stress, which is known as mitocytosis [17]. Migrasome-mediated mitocytosis is crucial for cellular health because it helps to maintain mitochondrial homeostasis under such stress. Metformin-loaded nanoparticles have been shown to enhance mitocytosis in chondrocytes, thereby improving mitochondrial quality and providing protection against osteoarthritis [7]. Nevertheless, the regulatory effect of metformin on mitocytosis in HUVECs remains unknown. It has been found that the migrasome core elements TSPANs are widely expressed in the cardiovascular system [44]. Although direct evidence is limited, we hypothesize that migrasome-mediated mitocytosis during cell migration contributes to the protective effects of metformin against SIPS in HUVECs.

In this study, we initially confirmed that metformin promotes the migration of HUVECs. This effect provides a cellular basis for enhanced migrasome production in endothelial cells. Migrasomes originate from retraction fibers at the trailing edge of migrating cells [42]. Retraction fibers serve as the structural scaffold for migrasome formation, with their number, branching, and length directly influencing the efficiency of migrasome production [45]. Using WGA staining, we observed that H_2_O_2_ inhibited the elongation of retraction fibers, whereas metformin promoted migrasome biogenesis in H_2_O_2_-stressed HUVECs. These observations underscore that metformin enhances migrasome biogenesis primarily by facilitating the formation and elongation of retraction fibers. Moreover, the co-localization of MitoSOX and WGA revealed that mitochondria enclosed within migrasomes were dysfunctional and exhibited high mtROS levels, suggesting that this process represents a selective quality control mechanism for clearing damaged mitochondria rather than nonspecific mitochondrial release. Furthermore, quantitative mtDNA analysis showed that metformin treatment did not lead to a significant change in extracellular mtDNA levels in the culture supernatant compared to the H_2_O_2_-treated group, which may be explained by the fact that migrasomes are primarily anchored to the substrate rather than being free in the culture supernatant [42]. However, mtDNA abundance within isolated migrasomes was moderately elevated in the metformin-treated group. These results raise the possibility that metformin may promote the selective packaging of damaged mitochondria into migrasomes. Collectively, these findings indicate that metformin facilitates the expulsion of damaged mitochondria under high oxidative stress via migrasome-mediated mitocytosis, thereby contributing to the maintenance of mitochondrial homeostasis in endothelial cells exposed to oxidative stress.

Consistent with the morphological observations, transcriptomic sequencing and protein expression validation supported a regulatory effect of metformin on mitocytosis. In HUVECs with SIPS, Western blot and immunofluorescence analyses demonstrated that metformin upregulated TSPAN4, an essential structural protein for migrasome biogenesis [46]. Metformin also restored DRP1 expression, which is thought to drive mitochondrial fission and facilitate the recruitment of damaged mitochondria into migrasomes [17]. The coordinated upregulation of TSPAN4 and DRP1 suggests that metformin modulates migrasome formation and mitochondrial dynamics to promote mitocytosis. Supporting this, transcriptomic analysis revealed that metformin-regulated DEGs were enriched in pathways related to cell adhesion, cytoskeletal regulation, and vesicle trafficking, indicating activation of membrane biogenesis and vesicle transport programs. Nevertheless, the precise mechanisms and interactions, including cytoskeleton remodeling, vesicle trafficking, and mitochondrial fission coupling associated with migrasome formation, remain to be further elucidated.

The AMPK pathway, a core sensor of cellular energy status, acts as a pivotal upstream regulator in mitochondrial quality control. AMPK participates in modulating mitochondrial dynamics and autophagic processes. Existing evidence has demonstrated that metformin can alleviate oxidative stress injury by activating autophagy through AMPK [47,48]. Our results showed that although metformin activated AMPK and autophagy, inhibition of AMPK did not completely reverse the effects of metformin, which is supported by the ROS level and Western blot analysis, suggesting the involvement of additional mechanisms in mitochondrial quality control. Regarding the link between AMPK and migrasomes or migrasome-mediated mitocytosis, the limited available evidence indicated that macrophage-derived migrasomes can transfer IL-1β to mesenchymal stem cells to activate the AMPK pathway, thus enhancing BMSC migration and promoting osteogenesis during fracture repair [49]. In our experiments, WGA and MitoSOX co-staining revealed that AMPK inhibition by Compound C (Met + Compound C) increased the abundance of free mitochondria but interfered with mitocytosis. We therefore cannot completely rule out a modulatory role of AMPK, and speculate that AMPK may be involved in the process of packaging damaged mitochondria into migrasomes, although the specific mechanism awaits further investigation. Collectively, the AMPK pathway and autophagy contribute to the metformin-mediated protective effects, with mitocytosis serving as one of the underlying mechanisms.

Given the critical role of TSPAN4 in migrasome formation, suppressing TSPAN4 expression is an effective strategy to reduce migrasome production and thereby interfere with migrasome-mediated mitocytosis [17,50]. In this study, we used siTSPAN4 to assess whether migrasome-mediated mitocytosis contributes to the protective effects of metformin. Morphological observations of migrasome formation and cellular phenotypic assays showed that TSPAN4 knockdown by siRNA largely abolished metformin’s benefits, including reductions in ROS, alleviation of SA-β-gal activity, and restoration of angiogenic capacity. These findings suggested that the protective effects of metformin require normal TSPAN4 expression and intact migrasome formation, supporting a potential role for migrasome-mediated mitocytosis in mediating metformin’s protective effects against SIPS in HUVECs. Although further investigation with temporal studies and specific mitocytosis inhibitors is still needed to fully distinguish the contribution of mitocytosis from potential direct antioxidant effects or other well-established mitochondrial quality control pathways, the present study, for the first time, provides preliminary evidence that metformin improves the migration and angiogenic ability of HUVECs under SIPS by maintaining mitochondrial homeostasis, at least partly through the enhancement of migrasome-mediated mitocytosis.

The present study still has several limitations. Firstly, all experiments in this study were carried out in vitro using a H_2_O_2_-induced SIPS model of HUVECs, which may not fully reflect the complex physiological and pathological microenvironment of the vascular endothelium. Thus, the protective effects of metformin on vascular endothelial cells via mitocytosis observed in this study need to be further validated in in vivo models. Moreover, our current exploration of the mechanisms underlying metformin-mediated regulation of mitocytosis focuses on morphologic changes and the expression of key associated molecules; the upstream signaling cascades driving metformin-induced migrasome biogenesis and the interaction between mitocytosis and other mitochondrial quality control mechanisms have not been investigated in greater depth. Further research is needed to elucidate the specific mechanisms by which metformin interacts with migrasome-mediated mitocytosis and to explore the therapeutic implications of this potential interaction. A comprehensive understanding of these mechanisms may yield novel insights into the broader cellular health benefits of metformin and its potential applications beyond diabetes management.

## 4. Materials and Methods

### 4.1. Source and Cultivation of HUVECs

Human umbilical vein endothelial cells (HUVECs) were purchased from ScienCell^TM^ Research Laboratories (Carlsbad, CA, USA) [51]. Cells were cultured in Dulbecco’s Modified Eagle Medium (DMEM; Gibco, Grand Island, NE, USA) supplemented with 10% fetal bovine serum (FBS; Biofiven, Guangzhou, China) and 1% penicillin/streptomycin (P/S; Gibco) at 37 °C.

### 4.2. Induction of HUVECs Senescence

Hydrogen peroxide (H_2_O_2_) was used to induce SIPS. HUVECs seeded into 6-well plates were exposed to H_2_O_2_ at concentrations of 0, 50, 100, and 200 μM for 2 h [52,53]. Cells were then washed with PBS and changed to fresh medium. After 24 h, the senescence-associated β-galactosidase (SA-β-gal) activity was assessed, and Western blot and qRT-PCR were applied to detect the protein and mRNA expression of senescence-related markers (p16 and p21) under different concentrations of H_2_O_2_ induction. Based on the SA-β-gal staining results and expression levels of senescence-related markers, 100 μM H_2_O_2_ was used to establish SIPS in HUVECs.

### 4.3. Live/Dead Assay

To assess the effect of metformin on the viability of HUVECs with SIPS, a Live/Dead cell staining kit (Procell, Wuhan, China) was employed. HUVECs were seeded in culture dishes at a density of 10^4^ cells/well. Metformin hydrochloride (Aladdin Biochemical Technology Co., Ltd., Shanghai, China; M107827, purity ≥ 97%) was dissolved in DMEM to a 200 mM stock solution. SIPS was induced in cells with 100 μM H_2_O_2_ for 2 h and then cultured with fresh complete medium (DMEM supplemented with 10% FBS and 1% P/S containing 0, 1, 5, 10, 20, 40 mM metformin) for 24 h. PI (7.5 μM) and Calcein-AM (1 μM) were added to label dead cells and live cells, respectively, according to the manufacturer’s protocol. The number of dead cells was examined with a CLSM (FV3000, Olympus, Tokyo, Japan) and measured using ImageJ (version 1.50, National Institutes of Health, Bethesda, MD, USA) from five random fields.

### 4.4. SA-β-Gal Staining

To evaluate the effects of metformin on the biological behaviors of HUVECs and mitochondrial-related function, HUVECs were divided into three groups: (1) HUVECs group (control group): HUVECs without any additional treatment; (2) HUVECs + H_2_O_2_ group: HUVECs treated with 100 μM H_2_O_2_ for 2 h to induce SIPS; (3) HUVECs + H_2_O_2_ + Met group: After H_2_O_2_ stimulation, HUVECs were cultured in fresh basal medium containing metformin for an additional 24 h.

SA-β-gal staining was applied to observe the effects of metformin with different concentrations on the SA-β-gal activity, according to the manufacturer’s protocol (Beyotime, Shanghai, China). HUVECs were treated with H_2_O_2_ and metformin as described above. Based on the biocompatible concentration determined by the Live/Dead assay, a concentration between 1~20 mM metformin was used in this assay. After fixation with 4% PFA, HUVECs were incubated with SA-β-gal staining solution at 37 °C for 24 h. Five random fields were captured by an inverted microscope (Zeiss Axio Observer, Oberkochen, Germany), and the proportions of SA-β-gal-positive cells were calculated using ImageJ.

### 4.5. Cell Proliferation Assays

EdU assay was conducted on HUVECs cultured in glass-bottom dishes. Cells were seeded at a density of 5000 cells per well. After 3 days, cell proliferation was labeled with 10 μM EdU (Beyotime) for 1.5 h according to the manufacturer’s instructions. After cell nuclei were stained with Hoechst33342, five random fields from each group were captured with a confocal laser microscope (FV3000, Olympus). The proportion of EdU-positive nuclei in each group was calculated using ImageJ.

### 4.6. Assessment of Migration Ability

To evaluate the migration ability, a scratch assay was performed. HUVECs were seeded into 6-well plates at a density of 2 × 10^5^ cells/well. At 30 h after H_2_O_2_ induction, an artificial scratch was created across the cell monolayers with 200 μL pipette tips. Afterwards, the medium was replaced with fresh culture medium consisting of 10 mM metformin and 2% FBS. The closure of the induced gaps due to cell migration was monitored and recorded with an inverted microscope (Zeiss Axio Observer) after 24 h.

### 4.7. Assessment of Angiogenic Ability

HUVECs in each group were treated with H_2_O_2_ and/or metformin as described above. Afterwards, cells were seeded in Matrix gel (Biosharp, Beijing, China)-preincubated 48-cell plates at a density of 8 × 10^4^/well and cultured at 37 °C. After 6 h, the tubular structures formed in the plates were observed under the inverted microscope. Quantitative analysis of tube formation was performed using ImageJ. Furthermore, a Western blot was used to detect the expression levels of angiogenesis-related markers including vascular endothelial growth factor (VEGF) and Angiopoietin-1 (Ang-1) in HUVECs.

### 4.8. Intracellular ROS Detection

For detection of intracellular ROS levels, cells were loaded with the DCFH-DA probe [5-(and-6)-chloromethyl-2′,7′-dichlorodihydrofluorescein diacetate, acetylester, DCFH-DA, Beyotime] at 10 μM and incubated for 20 min at 37 °C. Cells were washed with culture medium without FBS and then immediately analyzed with a CLSM (Zeiss, LSM 980, Oberkochen, Germany).

### 4.9. Measurement of Mitochondrial Function

The mitochondrial function of HUVECs was analyzed through mitochondrial morphology and mitochondrial membrane potential. HUVECs were seeded in 15 mm glass-bottom dishes and assigned to three groups as described above. The morphology of mitochondria was visualized via MitoTracker green dye (Meilunstar, Dalian, China) according to the manufacturer’s protocol. For MMP detection, cells were incubated with JC-1 staining working solution (Beyotime) at 37 °C in the dark for 20 min. Fluorescence images were captured with a confocal laser scanning microscope (ZEISS LSM980).

### 4.10. RNA-Sequencing and Bioinformatic Analyses

RNA sequencing was performed to further analyze the transcriptome profiles of HUVECs under SIPS with or without metformin. Total RNA was extracted from HUVECs (*n* = 3 per group) using Trizol reagent (Invitrogen, Thermo Fisher Scientific, Waltham, MA, USA). RNA quality was checked with an Agilent 2200 and stored at −80 °C. The cDNA libraries were constructed for each RNA sample using the VAHTS Universal V6 RNA-seq Library Prep Kit for Illumina (Vazyme, Inc., Nanjing, China) according to the manufacturer’s protocol, and sequenced via DNBSEQ-T7 (MGI Tech Co., Ltd., Wuhan, China) on a 150 bp paired-end run. Differentially expressed genes (DEGs) were analyzed with the following criteria: |Fold change| > 1.5 and false discovery rate < 0.05. GO analysis and KEGG analysis were performed to elucidate the biological implications and significant pathways of the DEGs.

### 4.11. Migrasomes and Mitochondria Staining

Migrasomes, a newly discovered organelle involved in mitocytosis, facilitate the selective removal of damaged mitochondrial components [42]. For migrasomes and mitocytosis visualization, HUVECs were seeded at a density of 2 × 10^5^ cells per well on 20 mm glass-bottom confocal dishes precoated with fibronectin (1 μg/mL). Upon reaching 60% confluency, cells were treated with H_2_O_2_ (100 μM) for 2 h, followed by metformin treatment. Wheat Germ Agglutinin (WGA; AAT Bioquest, Pleasanton, CA, USA) [54] and MitoTracker (Meilunstar) were applied to label migrasomes and mitochondria at final concentrations of 1 mM and 200 nM for 30 min, respectively. Additionally, to verify the status of mitochondria enclosed within migrasomes, MitoSOX (MCE, Monmouth Junction, NJ, USA) was co-stained with WGA following the manufacturer’s instructions. Finally, the cells were observed under a confocal microscope (ZEISS LSM980).

### 4.12. Detection of AMPK Activation and Mitophagy Involvement

It is widely known that metformin acts through AMPK-dependent pathways. To detect the role of the AMPK pathway and mitophagy in metformin-induced effects, the expression levels of AMPK, p-AMPK, PINK1, Parkin, and LC3B were analyzed via Western blot. Additionally, Compound C was applied to test whether inhibiting AMPK alters the effects of metformin. HUVECs were treated with H_2_O_2_ and incubated with Compound C (10 μM, 30 min) and metformin, alone or in combination. The expression changes in proteins associated with the AMPK pathway (AMPK, p-AMPK) and mitophagy (PINK1, Parkin, LC3B) were examined via Western blot. ROS levels were detected as described above. WGA and MitoSOX staining were applied to observe the alterations in migrasome formation and mitocytosis caused by AMPK inhibition.

### 4.13. Quantification of Extracellular Mitochondrial DNA

To determine whether mitochondria were actually expelled extracellularly, cell-free mitochondrial DNA (mtDNA) levels in the culture supernatant and isolated migrasomes were quantified. Conditioned medium was collected from each treatment group (500 μL per sample) and centrifuged at 2000× *g* for 10 min to remove cells and debris [55]. Migrasomes were isolated as previously described in our published work [50]. DNA was extracted using a QIAamp DNA Mini Kit (Qiagen, Hilden, Germany). Quantitative real-time PCR was performed to measure the levels of mitochondrial DNA (mtDNA) using primers for NADH dehydrogenase subunit I (ND1) and nuclear 18S rDNA. Relative mtDNA abundance was calculated and normalized to 18S rDNA.

### 4.14. Immunofluorescence Staining of TSPAN4

Since TSPAN4 is a core structural protein required for migrasome formation [56], immunofluorescence staining for TSPAN4 was performed to evaluate the involvement of migrasomes in the protective effects of metformin. After labeling with WGA for 30 min, cells were fixed with 4% PFA for 15 min, permeabilized with 0.5% Triton X-100 for 10 min (Beyotime), and blocked with 5% goat serum for 30 min (Boster, Wuhan, China). They were then incubated overnight at 4 °C with anti-TSPAN4 antibody (1:200, UpingBio, Hangzhou, China), followed by incubation with DyLight 555-conjugated secondary antibody (1:200, EMARbio, Beijing, China) for 50 min. After mounting with the DAPI-containing antifade medium (Beyotime), images were captured with a fluorescence microscope (Olympus FV3000).

### 4.15. Cell Transfection with siTSPAN4

As migrasome formation depends on TSPAN4 [56], small interfering RNA (siRNA) targeting TSPAN4 and a negative control were synthesized by Tsingke Biotechnology Co. (Beijing, China) to define the role of migrasome-mediated mitocytosis in metformin’s protective effects. The sequences of siTSPAN4 were listed in Table A1. HUVECs were transfected with 50 nM siRNAs using RNAiBoots Transfection Reagent (Yeasen, Shanghai, China), following the manufacturer’s instructions. The knockdown efficacy of siTSPAN4 was confirmed via qRT-PCR and Western blot. HUVECs transfected with the most effective siTSPAN4 were subsequently subjected to a series of functional assays. Transfected HUVECs were divided into the following groups: H_2_O_2_, H_2_O_2_ + Met, H_2_O_2_ + Met + si-NC, H_2_O_2_ + Met + si-TSPAN4. All groups were treated with 100 μM H_2_O_2_ for 2 h prior to subsequent assays.

### 4.16. Quantitative Real-Time PCR (qRT-PCR)

The expression levels of senescence-related genes (p16 and p21) and TSPAN4 were detected via qRT-PCR. Total RNA of HUVECs was extracted using the RNA-Quick Purification Kit (YiShan Biotech, Shanghai, China). cDNA synthesis was performed using a PrimeScript™ RT Master Mix (Takara Bio Inc., Kumamoto, Japan). qRT-PCR was conducted on a Light Cycler 96 Detection System (Roche, Basel, Switzerland) using the SYBR Green kit (AGbio, Changsha, China) according to the manufacturer’s protocols. GAPDH or β-actin served as the control for normalizing RNA expression levels. The primer sequences of the indicated genes are available in Table A2.

### 4.17. Western Blot Analysis

Western blot analysis was conducted to quantify the expression levels of senescence-related proteins (p16 and p21), angiogenesis-related proteins (VEGF and Ang-1), mitochondria dynamics-related proteins (DRP1), and migrasomes-specific proteins (TSPAN4). Cells were harvested and lysed in RIPA buffer (KeyGen BioTECH, Nanjing, China) supplemented with 1% protease inhibitor cocktail (CWBIO, Taizhou, China). After determining protein concentrations by the BCA assay kit (CWBIO), proteins from each group were separated by SDS-PAGE (ACE, Changzhou, China) and transferred to polyvinylidene fluoride (PVDF) membranes (Millipore, Billerica, MA, USA). The membranes were blocked with 5% skim milk and subsequently incubated overnight at 4 °C with primary antibodies—namely, anti-p16 (1:1000, Zenbio, Chengdu, China), anti-p21 (1:1000, Zenbio), anti-VEGF (1:1000, ABclonal, Wuhan, China), anti-Ang-1 (1:1000, Zenbio), anti-DRP1 (1:1000, Zenbio), anti-TSPAN4 (1:2000, UpingBio), anti-AMPK (1:1000, Affinity, Cincinnati, OH, USA), anti-p-AMPK (1:1000, Affinity), anti-PINK1 (1:800, UpingBio), anti-Parkin (1:1000, UpingBio), and anti-LC3B (1:1500, Abcam, Waltham, MA, USA)—followed by incubation with HRP-conjugated secondary antibody (1:5000, GNI, Tokyo, Japan) for 1 h at room temperature. Each experiment was performed in triplicate. Immunodetection was carried out using an enhanced chemiluminescence reagent (Abbkine, Wuhan, China) and visualized with a ChemiDoc imaging system (Bio-Rad, Shanghai, China).

### 4.18. Statistical Analysis

The SPSS 20.0 software (SPSS Inc., Chicago, IL, USA) was utilized for statistical analysis. Normal distribution was assessed using the Shapiro–Wilk test. Comparisons between two groups were conducted using independent unpaired two-tailed Student’s *t*-tests for normally distributed data. One-way analysis of variance (ANOVA) followed by Tukey’s test was performed to identify significant discrepancies among different groups, with statistical significance set at *p* < 0.05.

## 5. Conclusions

In conclusion, this work suggests that metformin alleviates early SIPS-associated changes in HUVECs by ameliorating mitochondrial dysfunction, in part through the modulation of migrasome-mediated mitocytosis. This study provides novel mechanistic insight into the vascular protective effects of metformin, and also sheds light on the potential role of mitocytosis in oxidative stress-induced endothelial cell dysfunction.

## Data Availability

The data presented in this study are available on request from the corresponding author Wei Zhao (zhaowei3@mail.sysu.edu.cn). RNA-seq data are deposited in Sequence Read Archive [https://www.ncbi.nlm.nih.gov/bioproject/PRJNA1439153] (accessed on 18 March 2026) [PRJNA1439153].

## Abbreviations

The following abbreviations are used in this manuscript:

SA-β-gal	Senescence-associated β-galactosidase
SIPS	Stress-induced premature senescence
HUVECs	Human umbilical vein endothelial cells
DMEM	Dulbecco’s Modified Eagle Medium
ROS	Reactive oxygen species
MMP	Mitochondrial membrane potential
TSPAN4	Tetraspanin 4
VAMP2	Vesicle-associated membrane protein 2
DRP1	Dynamin-related protein 1
VEGF	Vascular endothelial growth factor
Ang-1	Angiopoietin-1
FDR	False discovery rate
FC	Fold change
DEGs	Differentially expressed genes
ND1	NADH dehydrogenase subunit I
WGA	Wheat germ agglutinin
GO	Gene ontology
KEGG	Kyoto Encyclopedia of Genes and Genomes

## Appendix A

### Appendix A.1. siRNA Sequence

siRNA sequences of TSPAN4 are listed in Table A1.

**Table A1:** siRNA sequence.

	Forward Primer	Reverse Primer
*siTSPAN4#1*	GGCUUGCACCUGUACGGCA	UGCCGUACAGGUGCAAGCC
*siTSPAN4#2*	CGUGCUACGAGACGGUGAA	UUCACCGUCUCGUAGCACG
*siTSPAN4#3*	CCUACACGGACAAGAUUGA	UCAAUCUUGUCCGUGUAGG

### Appendix A.2. qRT-PCR Primer Sequence

Primer sequences applied in this study for qRT-PCR are listed in Table A2.

**Table A2:** qRT-PCR primer sequence.

	Accession No.	Forward Primer	Reverse Primer
*p* *16*	NM_000077	TGCCGAAGTCAGTTCCTTGT	CATTAGCGCATCACAGTCGC
*p* *21*	NM_001374512	GAGGCCGGGATGAGTTGGGAGGAG	CAGCCGGCGTTTGGAGTGGTAGAA
*ND1*	NC_012920.1	CCCTAAAACCCGCCACATCT	GAGCGATGGTGAGAGCTAAGGT
*18S*	NR_003286.4	TAGAGGGACAAGTGGCGTTC	CGCTGAGCCAGTCAGTGT
*TSPAN4*	NM_003271.5	GCTGTGGCGTCTCCAACTAC	CTTGGCAGTACATGGTCATGG
*β* *-actin*	NM_001101.5	CACCCTGAAGTACCCCATCGAG	GGATAGCACAGCCTGGATAGCA
*GAPDH*	NM_002046	GGACACTGAGCAAGAGAGGC	TTATGGGGGTCTGGGATGGA
